# Optical Inspection System for Gear Tooth Surfaces Using a Projection Moiré Method

**DOI:** 10.3390/s19061450

**Published:** 2019-03-25

**Authors:** Yi-Cheng Chen, Jr-Yi Chen

**Affiliations:** Department of Mechanical Engineering, National Central University; Taoyuan 32001, Taiwan; circle04617@gmail.com

**Keywords:** optical inspection, projection moiré, gear tooth topography

## Abstract

The demand for rapid online optical inspection of gear tooth surfaces is increasing, especially for precision gears. In this study, a non-contact optical measurement method was established for the inspection of gear tooth surfaces. For the system architecture, a halogen lamp was selected as the light source, and a collimated beam was produced by an autocollimator. Subsequently, moiré fringes were formed as the collimated beam went through the two linear gratings. The moiré fringes projected on the gear tooth surface were recorded with a charge-coupled device (CCD) camera, and the contour of the gear tooth surface was estimated and reconstructed from the phase information of the fringes by our developed computer codes. To verify the accuracy of the system, a spur gear tooth surface measured by a commercial coordinate measuring machine (CMM) was defined as the reference tooth profile. The tooth topography, involute profile deviation, and axial-direction deviation were successfully calculated by measuring the deviation of the optically measured surface based on the reference gear tooth profiles measured using the CMM.

## 1. Introduction

Gears are crucial components in the power transmission of automatic robotics and transportation vehicles. With the increasing demand for high-precision gears for speed reducers used in industrial robots and electric vehicles, high efficiency, as well as accurate in-situ inspection of the gear profile and quality grade, are essential in the gear production process. Moreover, the resolution required for high-quality gear profile inspection is in the range of a few micrometers. 

Conventionally, the gears are measured by contacting probes on the tooth surface with multiple points or by scanning several traces on the tooth surface. The industrial measurement facility for gear tooth surfaces is usually a coordinate measuring machine (CMM) with contact probing. The resolution increases with the increase of contact points on the tooth surface. However, the overall measuring time for a gear workpiece also increases with an increase in the number of measuring points. Therefore, the common practice is to measure only a few teeth, typically four teeth oriented 90 degrees apart. Moreover, only a few traces on one tooth surface are measured, including the involute profile and axial-direction traces. Consequently, these measurements present only partial information of all the gear teeth. 

Recently, optical metrology has been applied in measuring 3-D surfaces, including gear tooth surfaces. Optical metrology possesses the advantages of being a non-contact method and having high resolution and efficiency for workpiece contour measurement and surface quality inspection. Several methods exist for measuring optical topography, including the stereoscopic method, structured light, laser triangulation, conoscopic holography, the moiré method, and heterodyne interferometry [1,2,3,4,5,6,7,8,9,10,11,12,13].

The structured light method is becoming popular for handheld scanning devices that have relatively low precision but are more convenient. Peters et al. [1] applied structured light patterns for the high-efficiency measurement of gear tooth surfaces. A charge-coupled device (CCD) camera was used to capture the deformed stripes produced by the interaction between the light pattern and the tooth surface. Their measuring area was a few square centimeters, and the resolution was approximately 1 μm. 

Several different interferometric methods have been applied for 3-D contouring. Laser interferometry has high resolution but is sensitive to vibration and air turbulence during the phase-shifting process. Fang et al. [2] used laser interferometry to compensate for the inevitable installation errors that occur when measuring the gear tooth surface. Their experimental results demonstrated the feasibility of their compensation method. 

The laser triangulation technique is the most common method for online non-contact inspection; however, it has fundamental limitations of limited precision and short standoffs. In addition, laser triangulation is used to measure 3-D contours, especially for large workpieces that conventional CMM cannot measure. Smith et al. [3] used point laser triangulation for the inspection of helical gear. They studied an optimal path plan for helical gear profile inspection with a point laser triangulation probe to avoid collision and create a complex sensor-to-surface orientation. Recently, Auerswald et al. [4] proposed a laser line triangulation sensor for a large cylindrical involute gear with a pitch diameter of 922 mm and a face width of 246 mm. They developed a laser line triangulation sensor that can provide 1280 points at a 25-mm line width, and the speed can reach 200 lines/s. Their measurement deviation is ±8.2 μm due to random and systematic errors. The measuring speed is fast, but the accuracy can be improved. 

Conoscopic holography has the advantages of a high viewing angle, robustness, and a high point sampling rate. It has also been used for 3-D profile measurement with CMM [5] and in adverse environments [6]. Fernández et al. [5] integrated a conoscopic holography sensor onto a CMM and obtained an accuracy similar to that of laser triangular sensors. The errors of the conoscopic holography sensor compared with the CMM results range from 4 μm to 18 μm. Enguita et al. [6] developed an optical profilometer for distance calculation based on conoscopic holography and used it in a harsh steelmaking environment. The resolution was 0.2 mm with a long working distance of 1200 mm when they used conoscopic holography to inspect hot (over 800 °C) steel slabs. Álvarez et al. [7] reviewed the application of conoscopic holography techniques for submicron roughness measurement and high-temperature surface detection. They reported that multiple wavelength conoscopic holography can correctly resolve 3-μm steeps, extending the measurement range of the conoscopic holography to online roughness measurement and microdefect detection. Moreover, an online inspection system using conoscopic holography integrated with a robot was implemented for inspecting automobile synchronizer hubs. The 3-D data were acquired and measured automatically for every workpiece, and the precision was reported to nearly ±1 μm.

The moiré technique, which is similar to interferometry, has been applied in several moiré methods in 3-D surface contouring, including projection moiré, shadow moiré, and moiré with heterodyne interferometry [8,9]. Chang et al. [10] proposed a method for reconstructing surface profiles based on projection moiré and heterodyne interferometry. The projection grating moves at a constant velocity along the grating plane to produce a series of sinusoidal waves, similar to heterodyne interferometric signals. The resolution of this system is improved to approximately 1.9 μm by combining the projection moiré and heterodyne interferometry. 

Jeong and Kim [11] presented a high-speed moiré method by performing 3-D contouring with one fringe captured on the basis of the color grating projection. Practically, the accuracy of the moiré method can be affected by errors induced during phase-shifting. Mu et al. [12] investigated the error analysis of the phase-shifting in shadow moiré by changing the incidence angle of the collimating beams. Experiments were performed to verify the proposed technique, and the results were found to be 5% better than those of the conventional phase-shifting technique.

Sciammarella et al. [13] applied projection moiré in evaluating gear contours and reported the feasibility of using the projection moiré method. They produced the topographic information on a gear surface and compared it with that provided by a mechanical tactile device. The involute profiles of the gear measured by the optical method and by the mechanical method were examined and compared. However, the lead profile and 3-D topology errors, which are frequently presented in gear inspection report sheets, were not studied in their research.

In addition to the academic research on the optical metrology of gears, the industry has focused on developing commercial machines for the optical measurement of gear surfaces. Nikon announced the launch of HN-C3030 for 3-D gear surface measurement [14]. Laser structured patterns are projected on the gear tooth surface, and the computer numerical control (CNC) measurement machine has a five-axis movement. The resolution for this non-contact gear surface-measuring machine is reported to be 5 μm. Recently, Gleason announced the development of a gear rolling tester with laser inspection on a single machine [15]. The optical method for the profile inspection of cylindrical gears is reported to be ten times faster than the conventional probing method.

This study proposed an optical measurement method for the inspection of gear tooth surfaces. First, projection moiré fringes were formed on the gear tooth surface. Subsequently, the moiré fringe information produced by the five-step phase-shifting was used to determine the phase information to reconstruct the surface topography, and the contour of the gear tooth was estimated using our developed computer codes. To verify the accuracy of the inspection system, a spur gear tooth surface measured by a commercial CMM was defined as the reference tooth profile. The 3-D tooth topography, involute profile deviation, and lead deviation were successfully calculated by comparing the deviation of the optically measured surface and the reference gear tooth profiles using the CMM. Moreover, a gauge block was measured using our optical method to confirm measurement accuracy.

## 2. Projection Moiré Measurement System

### 2.1. Projection Moiré Fringes

Moiré fringes refer to a beat pattern formed by two gratings with approximately equal spacing. The precision depends on the numbers of fringes used in the measurement. For phase measurement, the surface height can be calculated as 1/100 of a fringe. 

For two straight line gratings, *G*_1_ and *G*_2_, the intensity transmission functions, *I*_*G*1_(*x*,*y*) and *I*_*G*2_(*x*,*y*), can be expressed as follows [8,16]:

(1) $$I_{G1}(x,y)=\alpha_{1}+\sum_{n=1}^{\infty }b_{1n}\cos[n\varphi_{1}(x,y)],$$

(2) $$I_{G2}(x,y)=\alpha_{2}+\sum_{m=1}^{\infty }b_{2m}\cos[n\varphi_{2}(x,y)]$$

where *φ*(*x*,*y*) is the function representing the basic shape of the grating lines; *α*_1_ and *α*_2_ are the average intensities; *b*_1*n*_ and *b*_1*m*_ denote the grating line profiles, such as square, triangular, and sinusoidal wares. Straight line gratings are used in this study; therefore, the above equations can be simplified and rewritten as follows:

(3) $$I_{G1}(x,y)=\alpha_{1}+b_{1}\cos[\varphi_{1}(x,y)],$$

(4) $$I_{G2}(x,y)=\alpha_{2}+b_{2}\cos[\varphi_{2}(x,y)]$$

When the two gratings are superimposed, the resulting intensity transmission function can be attained by the product of Equations (3) and (4):

(5) $$\begin{array}{c} I_{G1}I_{G2}=\alpha_{1}\alpha_{2}+\alpha_{1}b_{2}\cos[\varphi_{2}(x,y)]+\alpha_{2}b_{1}\cos[\varphi_{1}(x,y)]\\ +b_{1}b_{2}\cos[\varphi_{1}(x,y)]\cos[\varphi_{2}(x,y)], \end{array}$$

The first three terms of Equation (5) contain the information for the two patterns separately. The interesting term is the last term of Equation (5), and it can be expressed and rewritten as follows:

(6) $$\begin{array}{c} b_{1}b_{2}\cos[\varphi_{1}(x,y)]\cos[\varphi_{2}(x,y)]=\frac{1}{2}b_{1}b_{2}\cos[\varphi_{1}(x,y)+\varphi_{2}(x,y)]\\ +\frac{1}{2}b_{1}b_{2}\cos[\varphi_{1}(x,y)-\varphi_{2}(x,y)], \end{array}$$

where $\cos[\varphi_{1}(x,y)+\varphi_{2}(x,y)]$ is the high-frequency portion of the two superimposed gratings, whereas $\cos[\varphi_{1}(x,y)-\varphi_{2}(x,y)]$ is the low-frequency portion. Generally, the low-frequency term can be used to predict the moiré fringes and is observable by human eyes and optical sensors. 

If the two gratings form an angle of 2*α*, and the *y*-axis bisects the intersecting angle, the two grating functions can be rewritten as follows:

(7) $$\varphi_{1}(x,y)=\frac{2\pi }{\lambda_{1}}(x\cos\alpha +y\sin\alpha ),$$

(8) $$\varphi_{2}(x,y)=\frac{2\pi }{\lambda_{2}}(x\cos\alpha -y\sin\alpha ),$$

where *λ*_1_ and *λ*_2_ denote the grating period of the two gratings. Equations (7) and (8) can be rewritten as follows:

(9) $$\varphi_{1}(x,y)-\varphi_{2}(x,y)=\frac{2\pi }{\lambda_{b}}x\cos\alpha +\frac{4\pi }{\bar{\lambda }}y\sin\alpha ,$$

where $\bar{\lambda }$ = (*λ*_1_ + *λ*_2_) is the average grating period, and *λ_b_* = *λ*_1_*λ*_2_/(*λ*_1_ + *λ*_2_) is the beat wavelength between the two gratings.

According to Equation (6), the moiré fringes’ patterns can be lines whose centers satisfy the following condition:

(10) $$\varphi_{1}(x,y)-\varphi_{2}(x,y)=2M\pi ,$$

where *M* is an integer and represents the fringe order. Substituting Equation (9) into Equation (10) provides the following expression:

(11) $$\frac{2\pi }{\lambda_{b}}x\cos\alpha +\frac{4\pi }{\bar{\lambda }}y\sin\alpha =2M\pi ,$$

Furthermore, in this study, the two gratings have different periods and are parallel to each other, which leads to the second term of Equation (9) being zero. The moiré fringes will be lines that satisfy the following equation:

(12) $$M\lambda_{b}=x=M(\frac{\lambda_{1}\lambda_{2}}{\lambda_{1}-\lambda_{2}}),$$

Equation (12) demonstrates that the formed fringes will be equal-spaced lines parallel to the gratings lines. Consequently, the superimposition of two gratings’ lines is similar to the superimposition of two plane waves. Bright fringes form when the two waves are in phase, whereas dark fringes form when they are out of phase. The moiré fringes of two straight line gratings can determine the center of interference for fringes formed by interference from two plane waves. 

### 2.2. Five-Step Phase-Shifting

The phase-shifting technique was used for projection moiré fringes to attain quantitative height information. The errors in the phase-shifting technique include errors in phase-shifting, environmental disturbances, such as air turbulence and vibration, and the nonlinearity of the recorded intensities of the CCD detector [17]. The Hariharan algorithm is less sensitive to phase-shifting calibration errors compared with three-step and four-step phase-shifting algorithms. 

The five-step phase-shifting method proposed by Hariharan et al. [17,18] is used in this study. In this method, five measurements of the fringe intensities are conducted and recorded. The step for each phase-shift is *π*/2, and the phases corresponding to each step are −*π*, −*π*/2, 0, *π*/2, and *π*. After the fringes are captured, the intensity on each pixel of the sensor for the five steps can be expressed as follows:

(13a) $$I_{1}\left(i,j\right)=A\left(i,j\right)+B\left(i,j\right)\cos[\varphi \left(i,j\right)-\pi ]$$

(13b) $$I_{2}\left(i,j\right)=A\left(i,j\right)+B\left(i,j\right)\cos[\varphi \left(i,j\right)-\frac{\pi }{2}]$$

(13c) $$I_{3}\left(i,j\right)=A\left(i,j\right)+B\left(i,j\right)\cos[\varphi \left(i,j\right)]$$

(13d) $$I_{4}\left(i,j\right)=A\left(i,j\right)+B\left(i,j\right)\cos[\varphi \left(i,j\right)+\frac{\pi }{2}]$$

(13e) $$I_{5}\left(i,j\right)=A\left(i,j\right)+B\left(i,j\right)\cos[\varphi \left(i,j\right)+\pi ]$$

where (*i*,*j*) is the pixel coordinate, *I_K_*(*i*,*j*) represents the intensity at *K*^th^ phase shifting (*K* = 1~5), *A*(*i*,*j*) is the constant term, that is, the intensity bias, *B*(*i*,*j*) is the intensity amplitude, and *φ*(*i*,*j*) is the phase information to be determined at the measurement point.

After the five equations are solved (Equation (13a–e)) simultaneously, the phase at each measurement point can be calculated as an arctangent of a function of the intensities measured at the same measurement point at each phase shifting:

(14) $$\varphi \left(i,j\right)=\tan^{-1}\frac{2\left(I_{2}-I_{4}\right)}{2I_{3}{-I}_{5}{-I}_{1}}$$

Phase unwrapping is subsequently applied to remove the discontinuous 2*π* phase jumps. The unwrapped phase map of the measured profile is applied to determine the height quantitatively. 

**Figure 1:**
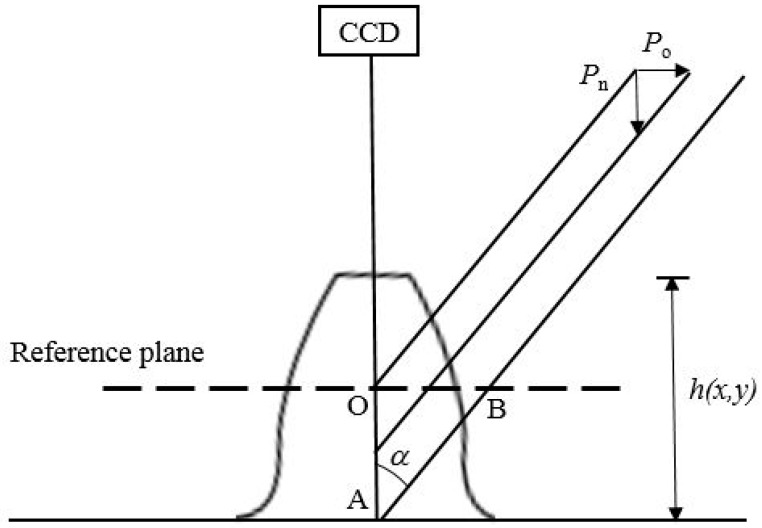
Illustration of the angle formed by the charge-coupled device (CCD) viewing direction and the projection moiré fringe, and a reference plane for the height estimation.

Figure 1 illustrates the projection moiré fringes on the gear tooth surface as viewed by a CCD camera. As demonstrated by Figure 1, a reference plane is placed at the middle of the tooth height, and an angle *α* is formed between the projection fringes and the viewing direction of the CCD camera. The surface height *h*(*x*,*y*) measured relative to the reference plane is as follows:

(15) $$h\left(x,y\right)=2\bar{OA}=\frac{2\bar{OB}}{\tan\alpha }=\frac{NP_{o}}{\tan\alpha }=NP_{n}$$

where *P_n_* is the contour interval (the height between adjacent contour lines in the CCD viewing angle), and *P_o_* is the spacing of the lines perpendicular to the CCD viewing direction. The fringe order N is the following:

(16) $$N=\frac{\varphi \left(x,y\right)}{2\pi }.$$

Moreover, the larger the angle *α* is, the smaller the contour spacing is. Theoretically, the sensitivity is maximized when angle *α* is 90°. By contrast, the measurement sensitivity approaches zero when angle *α* is near 0°. This case is not applicable in practice. However, a general guideline is that the angle chosen should be no larger than the maximum slope of the measured surface. In addition, shadows that lead to areas with missing data should be avoided, because the shadowed area cannot be contoured [17].

### 2.3. Experimental Framework

**Figure 2:**
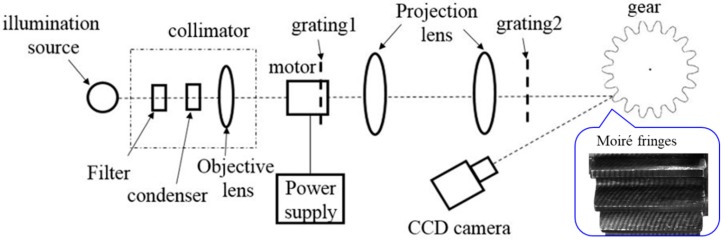
Illustration of the experimental framework of the projection moiré system.

**Figure 3:**
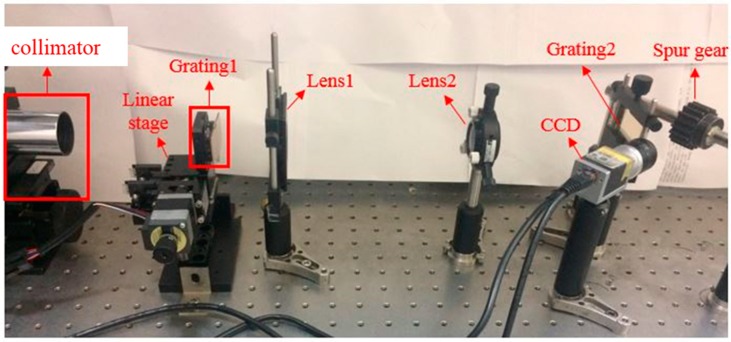
Photo of the experimental setup of the projection moiré system.

Figure 2 illustrates the framework of the proposed projection moiré technique established in this study, and Figure 3 displays the setup of the optical measurement system on the optical bench. As exhibited in Figure 2, a halogen lamp was used as the illumination source, and the collimated beam was produced by a collimator. As Figure 2 and Figure 3 illustrate, two projection lenses were used to enlarge the projected fringe period from grating 1, to form the moiré fringes with fringes projected from grating 2 on the tooth surface. Subsequently, the moiré fringes were built up on the gear tooth surface as the collimated beam passed through the two linear gratings. A linear stage driven by a servo motor enabled us to move grating 1 in the five-step phase-shifting. A CCD camera was used to capture the images of moiré fringes formed on the gear tooth surface. Table 1 summarizes the major components of the projection moiré system used in this study. 

**Figure 4:**
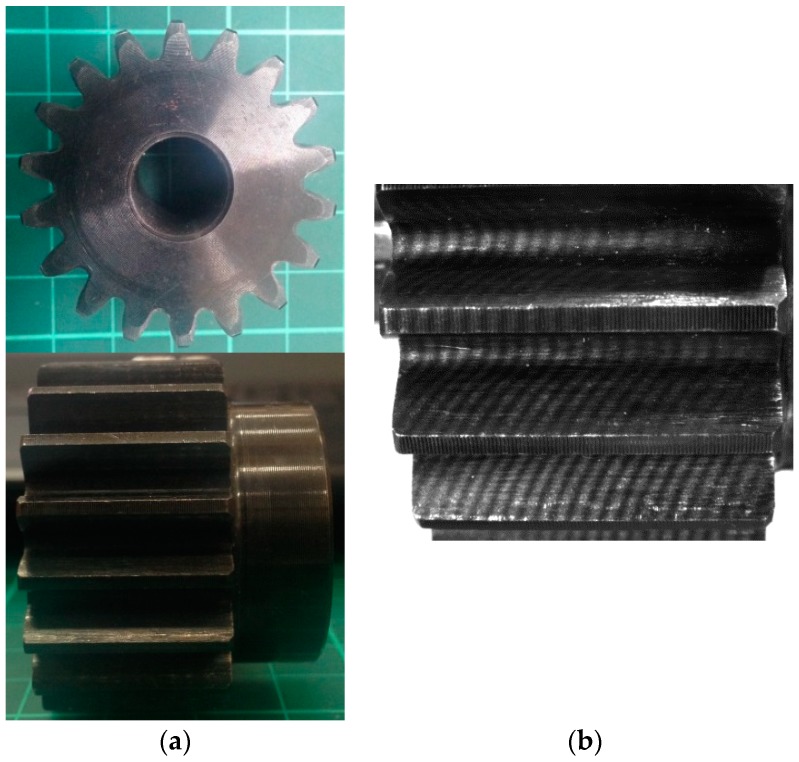
(**a**) Photo of the 18-tooth spur gear; (**b**) projected moiré fringes on the gear tooth surface.

Figure 4a illustrates the measured spur gear (KHK SS2-18), and its major design parameters are listed in Table 2. The module of the gear is 2 mm, its face width is 20 mm, and the whole tooth depth is 2.25 times that of the module (i.e., 4.5 mm). Thus, the area of one side of the tooth flank to be measured is approximately 20 mm by 2.25 mm. Figure 4b represents the projected moiré fringes on the gear tooth surface. The period of the moiré fringes displayed in Figure 4b is 806 μm. The projected moiré fringes are presented on three adjacent tooth surfaces, which implies that the optical method may inspect the three tooth surfaces in one measurement. Moreover, the incident angles of the projected fringes on the gear tooth surface and the viewing angle of the CCD camera have both been adjusted and tuned to minimize the erroneous phase information occurring on the tooth surface under inspection. Besides, the region of interest for inspection is only one tooth surface in this study.

### 2.4. GearTooth Surface Measurement Procedure

**Figure 5:**
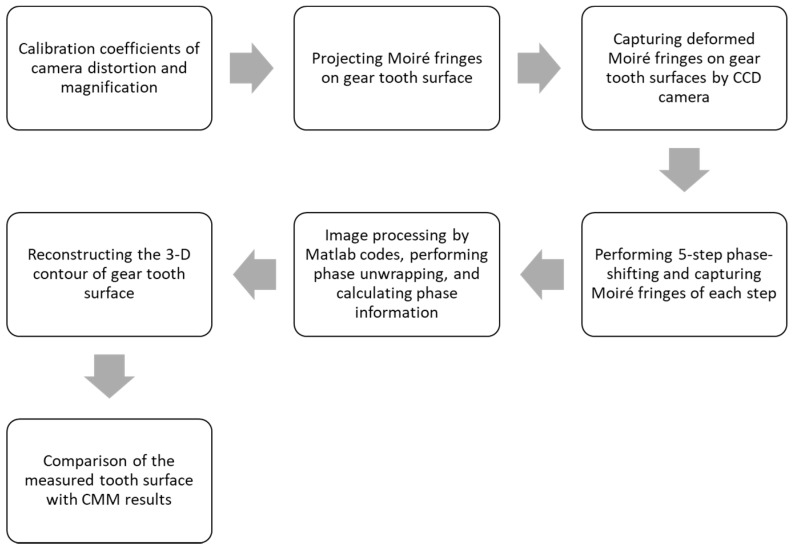
Process of the proposed optical inspection of the gear tooth surface.

**Figure 6:**
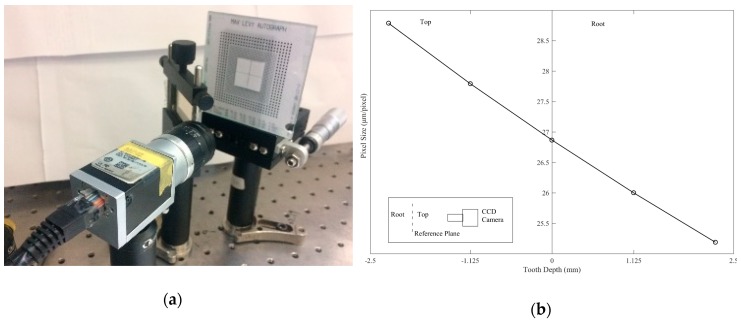
(**a**) Distortion coefficient correction with a grid distortion target; (**b**) calibration coefficient of the magnification along the tooth depth.

**Figure 7:**
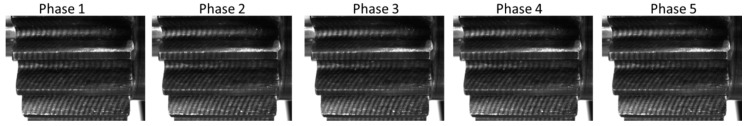
Moiré fringes on the gear tooth surface for each phase-shifting step.

**Figure 8:**
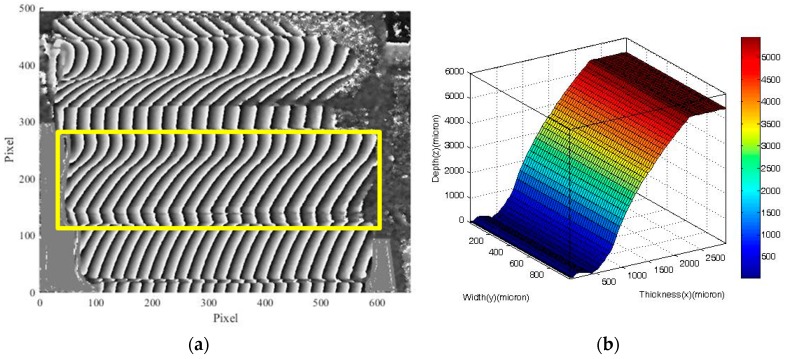
(**a**) Wrapped phase map on the gear tooth surface and the region of interest; (**b**) reconstructed 3-D tooth surface.

The measurement process of the spur gear tooth surface using the projection moiré method is illustrated in Figure 5. The process is described as follows:

(1) Perform the distortion correction of the CCD camera by obtaining the distortion correction coefficients with a grid distortion target, as illustrated in Figure 6a. Perform depth calibration to obtain the calibration coefficient of the magnification along the tooth depth, as illustrated in Figure 6b.
(2) Project the moiré fringes on the gear.
(3) Capture the deformed moiré fringes on the gear tooth surface.
(4) Perform the five-step phase-shifting by moving grating 1 on a linear stage and record the images of moiré fringes at each phase-shifting step. Because the fringe pitch is 806 μm, the translation of the grating for each phase-shifting is 201.5 μm. Figure 7 presents five images of moiré fringes on the gear tooth surface for each phase-shifting step.
(5) Perform the image processing and phase unwrapping of the images of moiré fringes using self-developed Matlab codes. Phase unwrapping is conducted using the branch-cut method, and the noise can be filtered using a Butterworth low-pass filter.
(6) Reconstruct the 3-D contour of the gear tooth surface from the calculated phase information according to the moiré fringes. Figure 8a shows the original phase on the tooth surface obtained from the five-step phase-shifting, whereas Figure 8b displays the reconstructed 3-D gear tooth surface.
(7) Compare and calculate the deviations of the optically measured surface from the CMM-measured reference surface.

**Figure 9:**
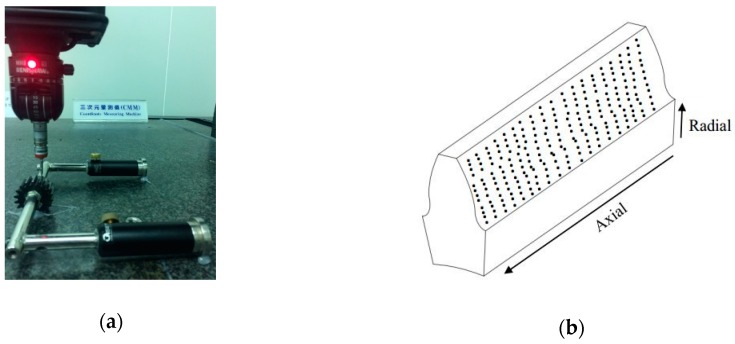
(**a**) Tooth surface measured by a commercial coordinate measuring machine (CMM); (**b**) measuring points on the tooth surface.

To compare and verify the measured results from the optical method, the gear tooth surface measured by a commercial CMM is used as a reference surface. A photo of the CMM measurement is presented in Figure 9a. The CMM is a Brown & Sharpe XCEL 7.6.5, and the accuracy of the CMM is 3.0 + 4 L/1000 μm, where L denotes the length of the measured feature. In addition, Figure 9b illustrates the radial and axial distribution of the measurement points on the gear tooth surface. The total measurement point number on one gear tooth surface is 2460 points. Curve fitting was applied to build the reference surface using the CMM for further calculation of the deviations from the optically measured surface based on the reference CMM-measured surface. 

## 3. Results

**Figure 10:**
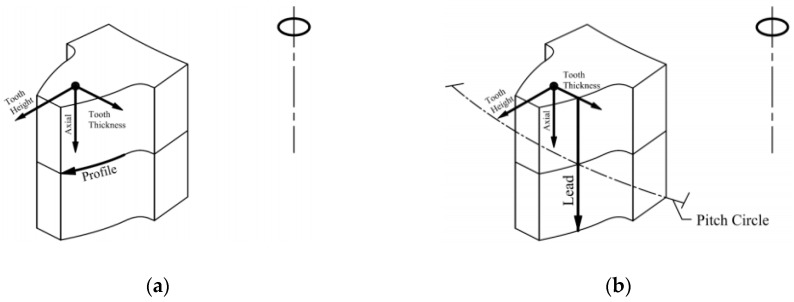
(**a**) Illustration of the involute profile measurement at the central cross-section; (**b**) illustration of the lead (axial) profile measurement on the pitch circle.

Generally, the data sheet of a gear inspection represents the quality of the gear tooth surface in three parts: (1) Involute profile deviation; (2) lead (axial-direction) deviation; and (3) topology deviation. The ideal profile of the cross-section of a gear tooth along the radial direction is an involute curve. Typically, the profile of the central cross-section of the gear blank is measured when the involute profile deviation is represented, as illustrated in Figure 10a. In addition, the profile along the lead direction is typically measured along the axial direction of the tooth surface on the pitch circle, as depicted in Figure 10b. Furthermore, a topology error represents a 3-D topology deviation of the measured real tooth surface from a reference tooth surface in a set of grid points. In this study, the determination of the three types of deviations was also developed using Matlab codes.

In this section, the tooth surface of the spur gear measured by the proposed optical method is compared with the CMM results, and the results of the three types of gear tooth surface deviations are calculated and represented thereafter. 

### 3.1. Involute Profile

**Figure 11:**
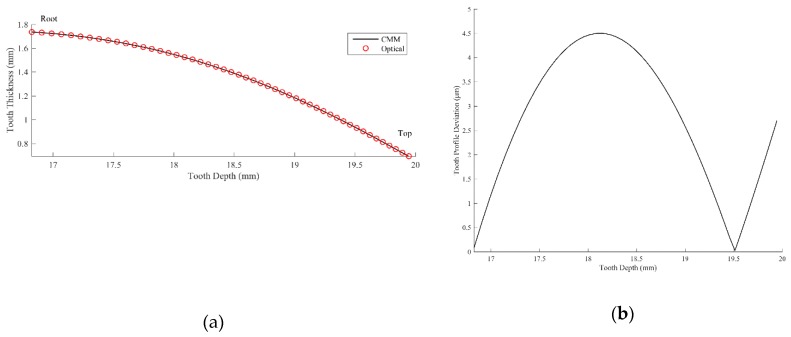
(**a**) Radial (involute) profiles measured by the CMM and optical system; (**b**) deviation of the involute profile of the projection moiré system from the CMM method.

Figure 11a depicts the radial tooth profile at the central cross-section of the gear blank measured by the optical projection moiré method and by the commercial CMM. The deviation of the profile measured by the optical method based on the CMM reference was calculated and is illustrated in Figure 11b. As demonstrated in Figure 11b, the mean value of the involute profile deviation is 2.67 μm and the maximum deviation is 4.49 μm. The average deviation of the optical measurement from the CMM measurement is within the accuracy of the CMM (i.e., within 3 μm), which implies that the proposed optical measurement method leads to reasonably accurate results compared with the commercial CMM. Furthermore, the optical method can inspect and measure the whole tooth surface in one measurement, whereas the CMM requires scanning or contacting the tooth surface in a point-by-point approach. Moreover, it is possible to contour the surface of several adjacent teeth from one measurement of the optical method. Therefore, the proposed projection moiré method exhibits comparable accuracy while saving time in the measurement of gear tooth surfaces.

### 3.2. Lead Profile

**Figure 12:**
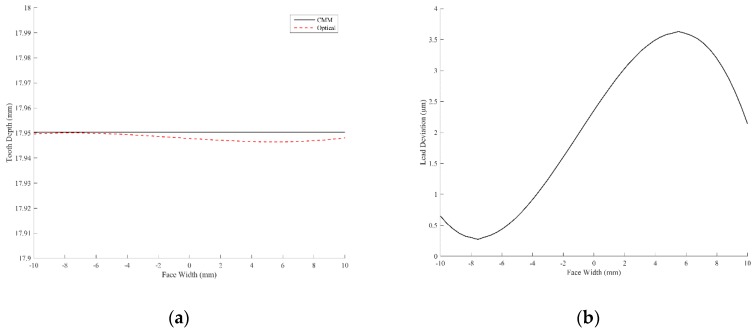
(**a**) Lead profiles measured by the CMM and optical system; (**b**) deviation of the lead profile measured by the projection moiré method from the CMM method.

The profiles along the lead (axial) direction on the pitch circle measured by the CMM and the optical method are presented in Figure 12a. The deviation of the lead profile measured by the optical method based on the CMM reference was calculated and is depicted in Figure 12b. As Figure 12b reveals, the maximum deviation along the lead direction is 3.63 μm, and the mean value of the deviation along the lead direction is 2.02 μm. The mean value of the deviation is within the accuracy obtained by the CMM. Therefore, the tooth surface measured by the projection moiré method is trustworthy and efficient and is comparable with the results obtained using a commercial CMM.

### 3.3. Tooth Surface Topology

**Figure 13:**
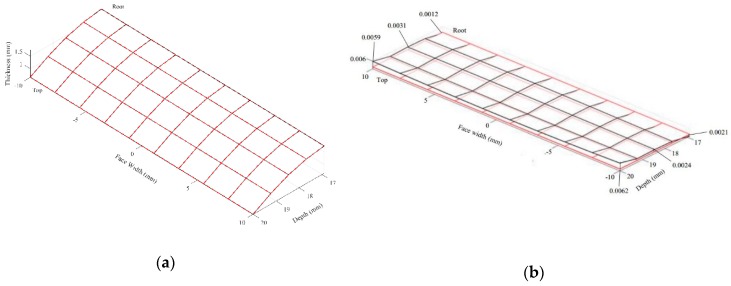
Spur gear tooth surface: (**a**) Topology of the gear tooth surface in 10 × 5 grids; (**b**) deviation of the tooth surface topology measured by the projection moiré system from the CMM method.

Figure 13a illustrates the 3-D gear tooth profile measured by the projection moiré method (black line) and the CMM (red line). The 3-D profiles from the two methods overlap because the deviations are only a few micrometers. The gear tooth surface is divided into 10 × 5 grids when representing the topology deviation. When the topology deviation is calculated, the tooth surface defined from the CMM measurement is considered as the reference surface. Subsequently, the normal (shortest) distance from each grid point on the reference surface to the surface measured by the optical method is calculated and recorded as the value of deviation at that grid point. Accordingly, the topology deviation of the surface measured by the projection moiré method from the CMM results is represented in Figure 13b. The mean value of the topology deviation is 2.81 μm, and the maximum deviation is 6.17 μm. 

## 4. Discussion

The proposed optical method can inspect the whole tooth surface of the spur gear, and the three types of deviations commonly used in the gear inspection reports are calculated and presented. Three forms of deviations of the gear tooth surface measured by the projection moiré method from the CMM results are illustrated in Figure 11, Figure 12 and Figure 13. Table 3 summarizes the maximum, minimum, mean value, and standard deviation of the three tooth surface deviations. The mean values of the tooth surface deviation of the three forms are all smaller than 3 μm, which is the accuracy range of the CMM used in this study. In addition, the standard deviations of the three types of deviations are all in the range of 1–2 μm. 

**Figure 14:**
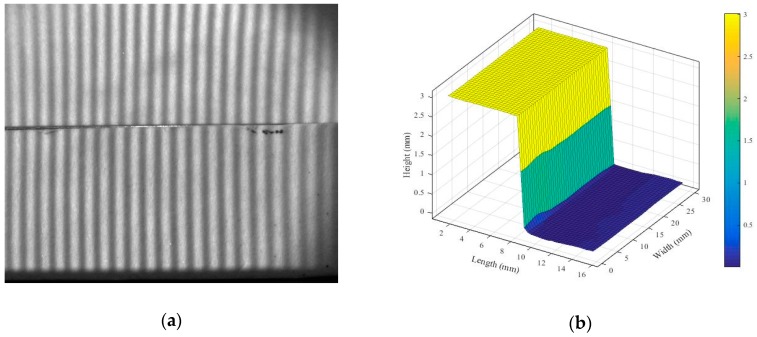

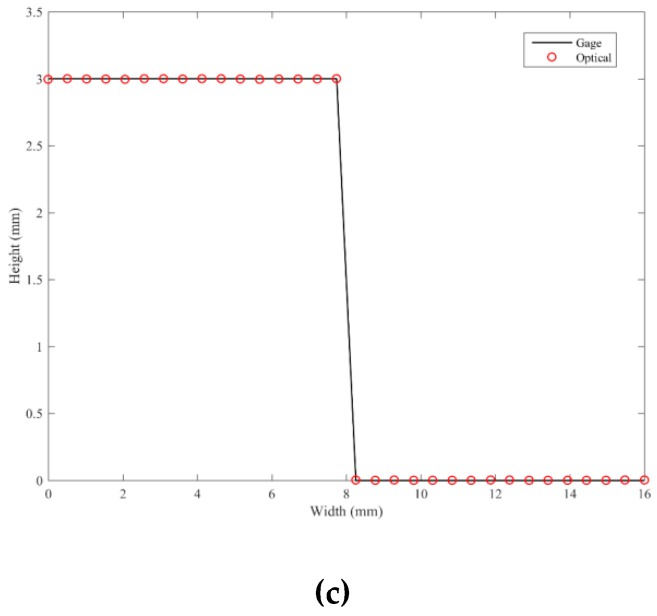
Gauge block measurements: (**a**) moiré fringes on the gauge block; (**b**) reconstructed 3-D profile; (**c**) optically measured profile and gauge block profile.

According to the experimental results presented in Table 3, the deviations of the three forms are all smaller than 3 μm, which is the accuracy range of the CMM. To determine the accuracy of our proposed method, a Mitutoyo grade K gauge block of dimension 30(L) × 8(W) × 3(H) mm^3^ was measured, and the deviation was calculated. Figure 14a,b depicts the moiré fringes on the gage block and the reconstructed 3-D profile by our optical method, respectively. In addition, Figure 14c illustrates the height reconstructed by our method and the nominal height of the gage block. The average value of the height deviation is 2.76 μm. 

Therefore, the accuracy of our optical measurement method is 2.76 μm, which is justified by the gauge block measurement and is also consistent with comparisons of the CMM and optical results in the preceding section. The accuracy of our system is comparable with the reported accuracy of 0.6–2.6 μm, and the average difference of 1.6 μm was confirmed by using a Zeiss CMM and following the method in [7]. The sources of errors in our system are discussed in the next paragraph. Moreover, the accuracy of our system can be further improved by modifying our projection moiré system.

The errors during the phase-shifting measurement may come from the following sources: (1) The data acquisition system; (2) environmental disturbance; (3) the nonlinearity of the sensors; (4) quantizing the signal from analog to digital; (5) source instability; (6) stray reflection. The data acquisition system includes the camera and the phase-shifting mechanism. A five-step phase-shifting algorithm was used in this study to minimize the effect of phase-shifting errors. Moreover, dial indicators and laser displacement sensors were applied to improve the accuracy of each phase-shifting step. The accuracy can be further enhanced by using a more accurate phase-shifting motorized stage, such as a piezoelectric actuator. 

Although the magnification calibration along the depth, as well as the distortion calibration of the camera, were performed for the system, the accuracy can be further improved if a telecentric camera lens system is used for image capturing. In addition, the vibration and air disturbance were kept to a minimum because the experiments were performed on a vibration-isolated optical bench in a laboratory with shielding curtains. Surface reflection and shadows should be avoided; otherwise, these may result in the saturation of sensor pixels and missing data on the corresponding measured area.

In summary, the proposed projection moiré method is considered a trustworthy and accurate non-contact method for gear tooth surface inspection. Moreover, the precision is related to the number of fringes used in the measurement; it can be further improved by applying different gratings to the projection moiré system. It will also be interesting and challenging to inspect two tooth surfaces simultaneously in the future, which may be affected by the shadowed area, reflection, and the steep slope of the tooth surface. The advantages of the projection moiré method are that it is contact-free, accurate, allows time savings, and that the tooth surface can be measured in one measurement. The optical method is expected to be applied to other 3-D surfaces as well, such as ball-screws, cams, screw compressors, and turbine blades.

## 5. Conclusions

An optical inspection method for gear tooth surfaces using the projection moiré is proposed and studied in this paper. The tooth surface of a spur gear was measured using the projection moiré method, and the measured results were compared with those from a commercial CMM. In addition, three forms of tooth surface deviations, namely involute profile, lead profile, and 3-D topology, measured by the optical method from the reference surface (CMM results) and presented in gear inspection sheets, were calculated and illustrated. The results revealed that the mean values of the profile deviations of the tooth surface measured by the proposed projection moiré method were within 3 μm, which was the accuracy range of the CMM used in this study. The accuracy of our optical measurement method is 2.76 μm, which was justified using the gauge block measurement. 

The results imply that the projection moiré method is an alternative, trustworthy, rapid, and non-contact method for measuring gear tooth surfaces. Furthermore, the precision grades of the measured gear, such as DIN (Deutsches Institut für Normung), ISO (International Organization for Standardization), and AGMA (American Gear Manufacturers Association) grades, can be determined and classified using a theoretical tooth surface geometry as the reference surface when calculating the involute, lead, and topology deviations. 

## Figures and Tables

**Table 1:** Components of the measurement system.

	Component	Specification
Projection module	Illumination	Halogen source
	Collimator	Trioptics 3-100-093
	Gratings (×2)	Pitch: 200 μm
	Biconvex lens (×2)	f = 75 mm
		f = 100 mm
Phase-shifting module	Stepping servo motor and driver	Oriental motor AR24SAK-1
	Linear stage	AFM-40-C5 Repeatability: ±2 μm
	Control system	LABVIEW codes
Image capture module	CCD sensor	Basler acA640-90gm
	Camera	Fujinon
	Image processing	MATLAB codes

**Table 2:** Design parameters of the measured spur gear.

Parameters	Values
Module (mm)	2
Teeth Number	18
Face Width (mm)	20
Pressure Angle (degrees)	20
Pitch Diameter (mm)	36
Addendum Diameter (mm)	40

**Table 3:** Summary of involute, lead, and topology deviations compared with the CMM results.

	Measured Tooth Surface Deviation (μm)			
	Mean	Maximum	Minimum	Standard Deviation
Involute profile	2.67	4.49	2.59 × 10^−2^	1.44
Lead profile	2.02	3.63	0.27	1.24
3-D Topology	2.81	6.17	0.41	1.31
